# Electroacupuncture Regulates Hippocampal Synaptic Plasticity via miR-134-Mediated LIMK1 Function in Rats with Ischemic Stroke

**DOI:** 10.1155/2017/9545646

**Published:** 2017-01-02

**Authors:** Weilin Liu, Jie Wu, Jia Huang, Peiyuan Zhuo, Yunjiao Lin, Lulu Wang, Ruhui Lin, Lidian Chen, Jing Tao

**Affiliations:** ^1^College of Rehabilitation Medicine, Fujian University of Traditional Chinese Medicine, Fuzhou, Fujian 350122, China; ^2^Fujian Key Laboratory of Rehabilitation Technology, Fuzhou, Fujian 350122, China

## Abstract

MircoRNAs (miRs) have been implicated in learning and memory, by regulating LIM domain kinase (LIMK1) to induce synaptic-dendritic plasticity. The study aimed to investigate whether miRNAs/LIMK1 signaling was involved in electroacupuncture- (EA-) mediated synaptic-dendritic plasticity in a rat model of middle cerebral artery occlusion induced cognitive deficit (MICD). Compared to untreatment or non-acupoint-EA treatment, EA at DU20 and DU24 acupoints could shorten escape latency and increase the frequency of crossing platform in Morris water maze test. T2-weighted imaging showed that the MICD rat brain lesions were located in cortex, hippocampus, corpus striatum, and thalamus regions and injured volumes were reduced after EA. Furthermore, we found that the density of dendritic spine and the number of synapses in the hippocampal CA1 pyramidal cells were obviously reduced at Day 14 after MICD. However, synaptic-dendritic loss could be rescued after EA. Moreover, the synaptic-dendritic plasticity was associated with increases of the total LIMK1 and phospho-LIMK1 levels in hippocampal CA1 region, wherein EA decreased the expression of miR-134, negatively regulating LIMK1 to enhance synaptic-dendritic plasticity. Therefore, miR-134-mediated LIMK1 was involved in EA-induced hippocampal synaptic plasticity, which served as a contributor to improving learning and memory during the recovery stage of ischemic stroke.

## 1. Introduction

Ischemic stroke results in a high mortality rate and increased disability rate all over the world [1]. Approximately 64% of stroke patients are often followed with cognitive impairment and 33% of them turn into dementia existing for several months during decubation [2]. Cognitive deficits arise frequently after ischemic stroke, which cause difficulties with analysis, concentration, organization, interpretation, and other abates in cognitive functions that bring about the low quality of life [3, 4]. The dysfunction of learning and memory is the cardinal symptom of cognitive impairment after stroke and is the main culprit of persistent sequelae [5]. A recent study demonstrated that the incidence rate of poststroke mild cognitive impairment was diagnosed in 24.4% of individuals after 3 years, and each year the mean growth rate is approximately 8% [6]. In addition to conventional cognitive training, electroacupuncture (EA) is a stretch therapeutic method of acupuncture, which is traditional acupuncture incorporation with modern electrotherapy. The clinical efficacy of EA on poststroke cognitive impairment has been widely demonstrated [7, 8]. However, the functional mechanism of EA is far from been fully elucidated.

The hippocampus is a pivotal structure of the brain; the area plays an important role in the formation of acquisition, consolidation, and recognition of declarative and spatial memory [9, 10]. The loss of hippocampal synapses and neurons in poststroke induces cognitive deficits including spatial reference learning and memory impairment [11, 12]. In the formation of spatial reference memory is closely related to the plasticity of dendritic spines and the morphological changes such as expansion and contraction [13]. Dendritic spines alter their shape to make the information spreading more easily and influence the synaptic efficacy (i.e., long-term potentiation and long-term depression) [14, 15], which have been widely considered as a cellular mechanism for learning and memory [16]. LIM domain kinase (LIMK1) is enriched in both axonal and dendritic growth cones of hippocampal pyramidal neurons in rats [17]. LIMK1 encodes a serine/threonine protein kinase that regulates the actin cytoskeleton by phosphorylating and inactivating the actin depolymerization factor (ADF)/cofilin [18]. In addition, LIMK1 is also known as having an important role in synapse and dendritic spine function. It has been reported that the knockout mice lacking LIMK1 are severely impaired in dendritic spine morphology and hippocampal long-term potentiation [19, 20]. Evidence showed that LIMK1 regulated long-term memory (LTM) and long lasting synaptic plasticity through interacting with and activating cyclic AMP response element-binding protein (CREB) [21].

In addition, a potential role for microRNAs (miRNAs or miRs) in synaptic function has been particularly intriguing given the evidence that a brain-specific miRNA contributes to synaptic development, maturation, and/or plasticity [22]. miRNAs are endogenous, noncoding RNAs that mediate the posttranscriptional regulation of gene expression mainly by binding to the 3′-untranslated region of messenger RNAs (mRNAs) [23]. A number of miRNAs have been isolated from nervous system, and a recent study has demonstrated a crucial role for dynamically regulating synaptic plasticity [24, 25]. Moreover, miRNAs have been implicated in hippocampus-dependent function, which have a significant potential in learning and memory formation, regulating LIMK1 expression to induce synaptic-dendritic plasticity [22]. Dendritic mRNAs encode diversified functionalities in hippocampal pyramidal neurons and play an important role in synaptic plasticity, as well as learning and memory [26].

Therefore, miRNA-LIMK1 can be considered as a target for cognitive deficit. Our previous study has shown that EA at Baihui (DU20) and Shenting (DU24) acupoints could improve cognitive impairment through Rho GTPases to enhance dendritic plasticity in rats with ischemic stroke [27]. Interestingly, it has been suggested that the activation of the Rho GTPases signaling is essential for LIMK1 activation by phosphorylation on threonine 508, which is widely known as master regulator of actin dynamics [28, 29]. Thus, the study aimed to elucidate whether EA at the DU20 and DU24 acupoints could improve the cognitive deficits in rats with ischemic stroke via miRNA-LIMK1-mediated synaptic plasticity to enhance spatial reference learning and memory.

## 2. Materials and Methods

### 2.1. Animal Ethics Statement

96 male Sprague-Dawley rats (weight, 250 ± 20 g) were obtained from Shanghai SLAC Laboratory Animal Co., Ltd. (Shanghai, China, SCXK2013-0005), and housed under pathogen-free conditions with a 12 h light/dark cycle. The study was approved by the Committee of Fujian University of Traditional Chinese Medicine (protocol #FUTCM-2015008) and was performed in accordance with the national guidelines for the care and use of laboratory animals. For euthanasia, 3% sodium pentobarbital (40 mg/kg body weight, i.p.) was used. Middle cerebral artery occlusion (MCAO) surgery was carried out under general anesthesia (1.5% isoflurane in 68.5% N_2_O and 30% O_2_). All efforts were made to minimize suffering.

### 2.2. Experimental Procedures

The MCAO-induced cognitive deficit (MICD) model was established as previously described [5, 30]. Rats were randomly assigned to four groups according to the random number table (*n* = 24 rats each group) as follows: (1) Sham group, (2) MICD group, (3) MICD+EA group, and (4) MICD+non-EA group. The left middle cerebral artery was occluded by a 4-0 nylon monofilament (0.23 mm in diameter, Jialing-bio, China). Focal cerebral ischemia was monitored using transcranial temporal laser Doppler (BIOPAC Systems, Goleta, CA, USA) and an 80% decrease in blood flow after the occlusion was recorded. After 90 min of occlusion, reperfusion was achieved by pulling out the filament to restore blood flow. Sham-operated rats of the Sham group underwent the same procedure, but arterial occlusion was not performed.

After 24 hours of MCAO surgery, rats of the Sham group and the MICD group received no treatment. Rats of the MICD+EA group were given EA treatment for 30 min per day for 14 consecutive days. The EA needles (diameter, 0.3 mm, needle purchased from Hualun acupuncture of Suzhou Co., Ltd., Suzhou China) were inserted at a depth of 2-3 mm into the Baihui (DU20, located in the median of frontalis) and Shenting (DU24, located in the median of the parietal bone) acupoints [31]. Stimulation was then generated using the EA apparatus [Model G6805, Shanghai Huayi (Group) Company, Ltd., Shanghai, China] and the stimulation parameters were set as follows: dilatational waves of 1~20 Hz (adjusted to the muscle twitch threshold), peak voltage of 6 V, and 0.2 mA intensity [31]. Rats of the MICD+non-EA group were given EA treatment at the bilateral nonacupoints (located in the costal region and 10 mm distal to the iliac crest) for 30 min per day for 14 consecutive days [32]. The EA needles and stimulation parameters of the bilateral nonacupoints were consistent with the EA treatment. Manipulators were experienced and blinded to the rat's group.

### 2.3. Behavioral Assessment

**Figure 1:**
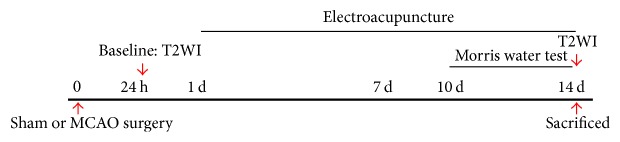
Experimental design used in the study.

Behavioral testing was conducted by researchers who were blinded to the rat's group. At 10 days after EA, all the rats were subjected to the Morris water maze test (Shanghai Xinruan Information Technology Co., Ltd, Shanghai, China) to evaluate spatial reference learning and memory (Figure 1). The Morris water maze consisted of a circular pool (diameter 150 cm, height 60 cm) filled with water (depth of 30 cm and temperature of 25 ± 2°C). A circular escape platform (diameter 12 cm, height 29 cm) was submerged 2 cm below the water surface, in the middle of the third quadrant of the pool and the reference objects around the pool were placed. Morris water maze tasks mainly include orientation navigation and space exploration trials. During the first set of trials, each rat was placed in the water at each of the four equidistant locations to the platform. When the rats arrived at the platform within the 90 sec time restriction and remained on it for 3 sec, they were considered to have found the platform and were scored by the time taken/length of the route. When the rats were unable to find the platform within 90 sec, they were placed on the platform for 10 sec and the time score was 90 sec. The time taken and the length of the route by which each rat found the safe platform were recorded by the computer. The average of the time taken and the length of the route for the four quadrants as a result of each rat were assessed every day. The duration of the first set of trials was performed on Days 10 to 13 after EA. The second part of the experiment was performed on Day 14, to examine the time in which rats found the location of the platform within the 90 sec time restriction, which tested their ability to remember the position of the platform. After all trials, the rats were dried thoroughly with a hair drier and returned to their cages (Guangzhou RiboBio Co., Ltd., Guangzhou, China). Morris water maze test was repeated three times.

### 2.4. Measurement of Brain Lesions

Animal MRI scans were performed on a 7.0 T MRI scanner (70/20 USR BioSpec, Bruker Biospin Gmbls, Germany) using a 38 mm birdcage rat brain quadrature resonator for radiofrequency transmission and reception. Animals were anesthetized with isoflurane/O_2_ (with 3% induction for 5 min and 1.2–1.5% maintenance in order to let the rats in the depth of anesthesia state) and kept warm with circuit. After anesthesia, the rat was put in prone position on a custom-made holder to minimize head motion, set the location of head position, and perform real-time monitor of the breathing rate and maintained in 40 breaths/min. Rat's temperature is maintained at 33 ± 2°C in the process of scanning holder to minimize head motion while respiration was maintained.

T2-weighted imaging (T2WI) in three planes with a fast spin echo (FSE) pulse sequence was first acquired to control rat head positioning. T2WI scan was acquired using a Rapid Acquisition with Relaxation Enhancement (RARE) pulse sequence with the following parameters: field of view = 32 mm × 32 mm, matrix size = 256 × 256, repetition time (TR) = 4200 ms, echo time (TE) = 35 ms, slice thickness = 1.0 mm, and slice gap = 0 mm.

Image J analysis and processing system was applied for T2W images, the percentage of the brain lesions = brain lesions volume/whole brain volume ×100%.

### 2.5. Golgi Staining

Golgi staining of brain was performed using an FD Rapid GolgiStain Kit following the manufacturer's instructions (FD Neurotechnologies, Inc., Columbia, MD, USA). The removed rat brains were placed incubated in a mixture of A and B solutions from the kit and stored in the dark at room temperature, following which they were transferred into solution C from the kit, stored at 4°C for 7 days. Finally, brains were then frozen and coronal sections (150 *μ*m) were made using a cryostat (Leica CM3050S, Leica Microsystems K. K., Tokyo, Japan). The sections incubated in a mixture of D and E solutions from the kit and then dehydrated in alcohol (50, 70, 90, and 100% for 5 min each), cleared in xylene, and were cover-slipped. The images finally were viewed under a microscope (Leica DM6000 B, Leica Microsystems, Wetzlar, Germany). The slides were reviewed by two or three pathologists blind to the study.

### 2.6. Transmission Electron Microscopy

Four rats in each group were anesthetized and the left ventricle was perfused with 200 mL of saline followed by 400 mL 4% paraformaldehyde (pH 7.4). The tissue was taken from the left ischemic hippocampus, cut into 1 mm^3^ size cubes, and fixed in 1% paraformaldehyde with 1% lanthanum nitrate tracer for 24 h followed by fixation with 3% glutaraldehyde for 24 h. Samples were fixed in 1% osmium tetroxide for 2 h and dehydrated in graded ethanol 1% lanthanum nitrate tracer (LNT) solution and embedded in araldite. Ultrathin hippocampal CA1 slices were obtained (90 nm); then they were stained with uranyl acetate and lead citrate and observed under TEM (H-7650; Hitachi, Ltd., Tokyo, Japan). The photos were obtained on hp digital CCD camera (SIS4 million voxel). Images were acquired digitally from a randomly selected pool of 10 to 15 fields under each condition.

### 2.7. Western Blotting

Isolated left hippocampus tissue was lysed in 100 *µ*L radioimmunoprecipitation assay (RIPA) buffer (Beyotime, Haimen, Jiangsu, China) plus protease inhibitors. Total protein (50 *µ*g) was loaded into 10% SDS-PAGE gels, electrophoresed, and then transblotted onto polyvinylidene difluoride membranes (Immobilon-P; Millipore, Billerica, MA) in a Tris-glycine transfer buffer. After being blocked in 5% milk in PBST for 1 h at room temperature with shaking, membranes were incubated with antibodies overnight at 4°C against the following: anti-LIMK1 antibody (dilution, 1 : 1000; ab81046, Abcam) and anti-LIM kinase 1 antibody (phospho-Thr508) (dilution, 1 : 1000; ab131341, Abcam) and *β*-actin (dilution, 1 : 1000; ab189073; Abcam). The following day, membranes were incubated in 5% milk (in TBST) with an anti-goat or anti-mouse IgG antibody (dilution, 1 : 5000; PerkinElmer Life Sciences, Waltham, MA) for 1 h at room temperature with shaking. Membranes were washed a minimum of four times (10 min per wash) in PBST between each antibody treatment. Detected bands were visualized using enhanced chemiluminescence and images were captured using a Bio-Image system (Bio-Rad Laboratories, Inc., Hercules, USA). Western blotting was repeated three times.

### 2.8. Real Time Quantitative RT-PCR

The expression of miRNAs was determined by real time quantitative reverse transcription polymerase chain reaction (RT-PCR). Total RNA was extracted from hippocampal region of the ipsilateral lesion tissue using the TRIzol Reagent (Life Technologies (AB & Invitrogen), Carlsbad, USA). Then extracted total RNA was reverse transcribed to generate cDNA according to manufacturer instructions of Revert AidTM First-Strand cDNA Synthesis Kit (Thermo Fisher Scientific, Beijing, China). The reverse transcription reaction was amplified using a Bio-Rad CFX96 Detection System (Bio-Rad, Hercules, CA, USA) with the Plexor™ One-Step qRT-PCR System (Promega, Madison, WI, USA). For miRNA amplification, the following primers were used: rno-miR-134 (RmiRQP0168, GeneCopoeia, Guangzhou, China). The fold change in relative mRNA and miRNA expression was determined using the 2^−ΔΔCt^ method as described previously [33] and U6 snRNA (RQP047936, GeneCopoeia) as mRNA and miRNA internal control, respectively.

### 2.9. Statistical Analysis

Statistical analysis was performed with the SPSS package for Windows statistical analysis software (Version 18.0, SPSS, Inc., Chicago, IL, USA). The data from all groups were determined by one-way analysis of variance (ANOVA) and Student's *t*-tests. The homogeneity of variance was analyzed using the least significant difference method and missing variance using the Games-Howell method. Intergroup comparisons of the brain lesions volume at different time points were performed with paired-samples *t*-tests. All data are presented as mean ± standard error, and the significance was regarded as at least *p* < 0.05. All final results were analyzed in a blinded manner.

## 3. Results

### 3.1. EA Improved Cognitive Impairment in MICD Rats

**Figure 2:**
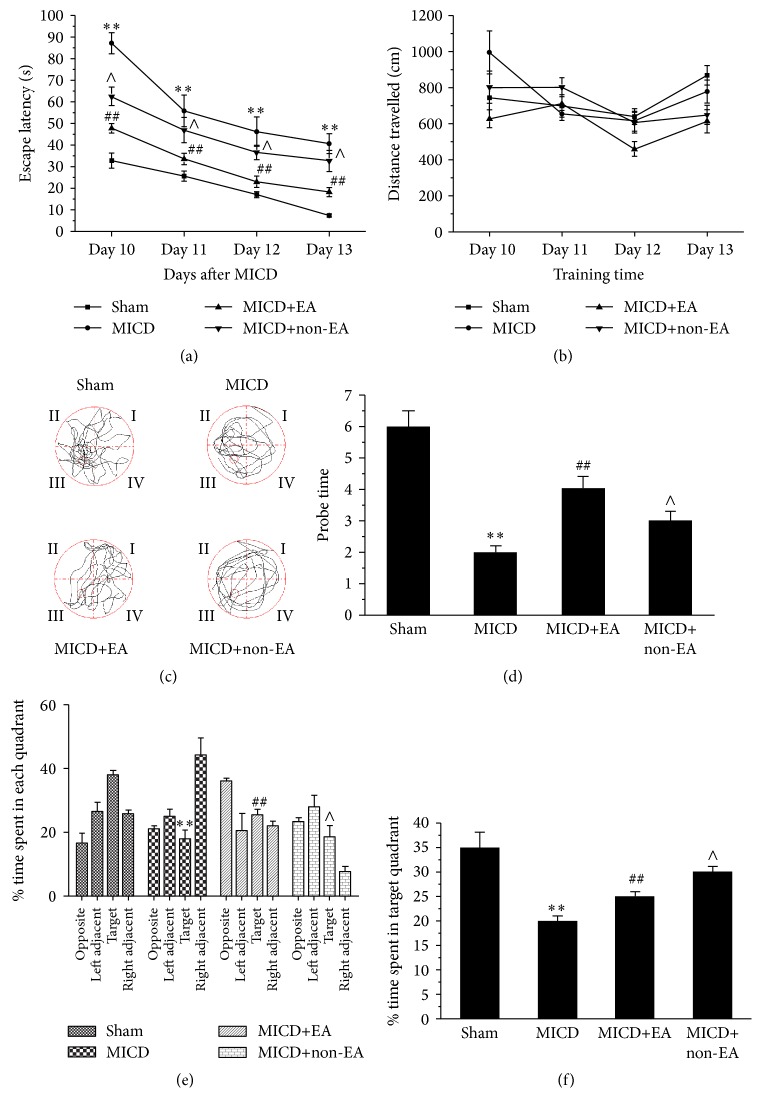
Evaluation of spatial reference learning and memory by Morris water maze test at Days 10–14 after EA. (a and b) Mean escape latency time and path length during the orientation navigation test on Days 10–13 after EA treatment. (c and d) Times the rats crossed over previous platform location on Day 14 after EA treatment during the spatial memory test in different groups. (e) The percentage of times spent in each quadrant in all probe trials is shown on Day 14 after EA treatment. (f) The times of passing the hidden platform position on Day 14 after EA treatment (each group; ^*∗∗*^ *p* < 0.01 versus the Sham group; ^##^ *p* < 0.01, ^#^ *p* < 0.05 versus the MICD group; ^∧∧^ *p* < 0.01, ^∧^ *p* < 0.05 versus the MICD+EA group). EA: electroacupuncture. MICD: middle cerebral artery occlusion induced cognitive deficits.

To assess the effect of EA on spatial reference learning and memory impairments in MICD rats, the Morris water maze (MWM) test was performed. All groups of rats learned to find the platform, and the latency time to reach the platform was reduced in the four training days. However, learning ability was significantly reduced in MICD rats compared with the Sham group (*p* < 0.01, Figure 2(a)); the MICD rats treated with EA significantly took less time to find the platform compared with the MICD group and the MICD+non-EA group (*p* < 0.01 or *p* < 0.05, Figure 2(a)). There was no significant difference about path length among the four groups (*p* > 0.05, Figure 2(b)). As illustrated in Figures 2(c) and 2(d), tracing images from the MWM test showed that in the space exploration test where the platform was removed the MICD rats passed through the original position of the platform fewer times than the Sham group (*p* < 0.01, Figure 2(d)), whereas in the MICD+EA group the times where the rats crossed the position of the platform were significantly increased compared with the MICD group and the MICD+non-EA group (*p* < 0.01, *p* < 0.05, Figure 2(d)). As illustrated in Figures 2(e) and 2(f), the percentage of time spent in the target quadrant was used for statistical analysis during the probe trial. The date showed that the rats spent more time in the target quadrant compared to other quadrants (*p* < 0.01; Figure 2(e)). The MICD group spent less time in the target quadrant compared with the other groups (*p* < 0.01; Figure 2(e)). The MICD+EA group spent more time in the target quadrant compared with the MICD group and MICD+non-EA group (*p* < 0.01, *p* < 0.05, Figure 2(f)). Therefore, these results suggest that acquisition or retention of spatial reference learning and memory was ameliorated in MICD rats by EA treatment.

### 3.2. EA Attenuated Left Cortex, Hippocampus, Corpus Striatum, and Thalamus Lesions in MICD Rats

**Figure 3:**
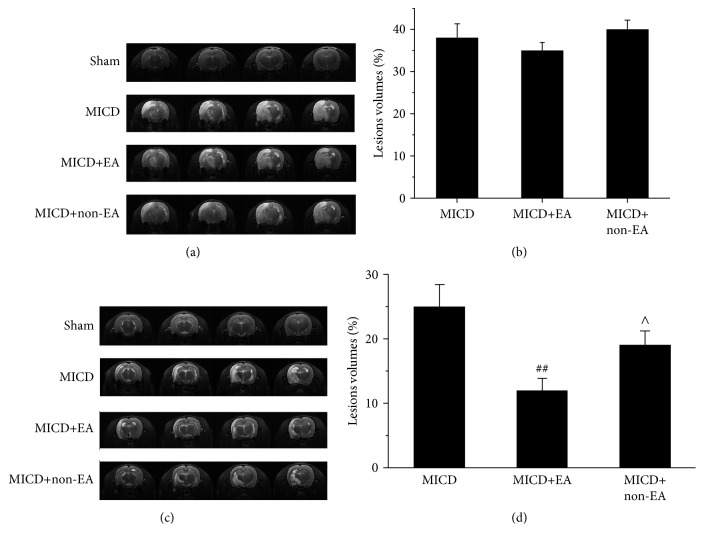
T2WI signal changes before and after EA (a and c). The brain lesions were measured by T2WI (slices from 11 to 13) in the Sham group, the MICD group, the MICD+EA group, and the MICD+non-EA group before (a) and after (c) EA. (b and d) Lesion volume is represented as a percentage of the total brain volume and data are presented as the mean ± standard deviation from 12 individual rats in each group. (^*∗∗*^ *p* < 0.01 versus the Sham group; ^##^ *p* < 0.01, ^#^ *p* < 0.05 versus the MICD group; ^∧∧^ *p* < 0.01, ^∧^ *p* < 0.05 versus the MICD+EA group). T2WI: T2-weighted magnetic resonance imaging; MICD: middle cerebral artery occlusion induced cognitive deficits.

The brain lesions were determined by T2-weighted imaging (T2WI) (Figures 3(a) and 3(c)) before and after EA treatment. The volumes of brain lesions that included left cortex, hippocampus, corpus striatum, and thalamus regions in the all groups had no significant difference before EA treatment (*p* > 0.05, Figures 3(a) and 3(b)). There was mild spontaneous recovery for 14 days in the brain lesions of the MICD group. The volumes of left cortex, hippocampus, corpus striatum, and thalamus lesions showed comprising approximately 23% of the whole brain in the MICD group, whereas the brain lesions sizes were significantly reduced to 15% in the MICD+EA group (*p* < 0.01, Figures 3(c) and 3(d)). The difference of brain lesions between the MICD+EA and the MICD+non-EA group showed significant changes at Day 14 after EA (*p* < 0.05, Figures 3(c) and 3(d)).

### 3.3. EA Increased the Density of Dendritic Spines in the Hippocampus of MICD Rats

**Figure 4:**
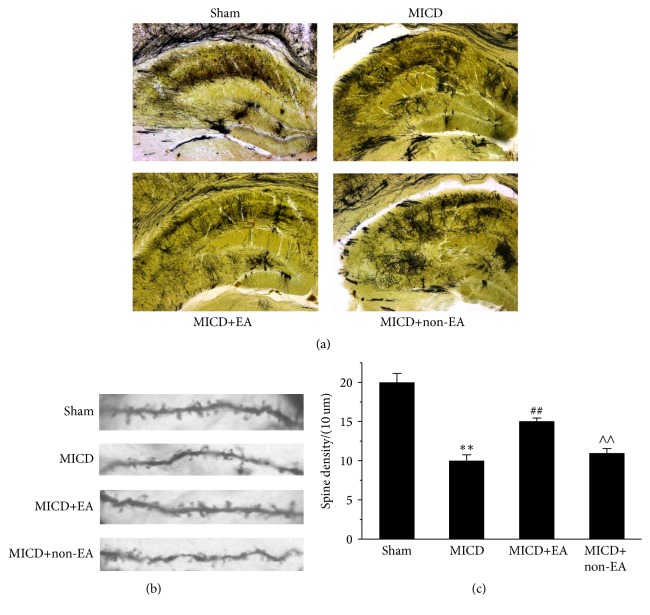
The structures and densities of dendritic spine in the hippocampus of rats. (a) Representative images of dendritic spine in the hippocampus of each group (Golgi staining, ×50). (b) Representative images of dendritic spine density and morphology from the pyramidal cell layer of hippocampal CA1 area in each group (Golgi staining, ×1000). (c) The density of dendritic spine was analyzed in hippocampal CA1 pyramidal cells in each group (*n* = 6/group; ^*∗∗*^ *p* < 0.01 versus the Sham group; ^##^ *p* < 0.01, ^#^ *p* < 0.05 versus the MICD group; ^∧∧^ *p* < 0.01, ^∧^ *p* < 0.05 versus the MICD+EA group). All experiments were repeated three times.

To investigate the function of EA on synaptic plasticity, dendritic spine density in the hippocampal neurons was analyzed by the primary basilar dendrites of Golgi-stained pyramidal neurons at Days 14 after EA. Golgi-Cox staining clearly filled the dendritic shafts and the spines of neurons from pyramidal neurons (Figure 4(a)). Hereby, the representative hippocampus showed that the density of dendritic spines was reduced in different degree by macroscopic examination in the MICD group, the MICD+EA group, and MICD+non-EA group, and the loss of dendritic spines in hippocampal CA1 was obvious in the MICD group. Thus, as illustrated in Figures 4(b) and 4(c), the density of selected dendritic spines that derived from hippocampal CA1 was significantly decreased in the MICD group compared with the Sham group (*p* < 0.01); however, the density of dendritic spines of the hippocampal CA1 in the MICD+EA group was more than that of the MICD group and the MICD+non-EA group (*p* < 0.01). In brief, EA treatment triggered large-scale remodeling of dendrites in the hippocampal area CA1.

### 3.4. EA Enhanced the Number of Hippocampus CA1 Synapses in MICD Rats

**Figure 5:**
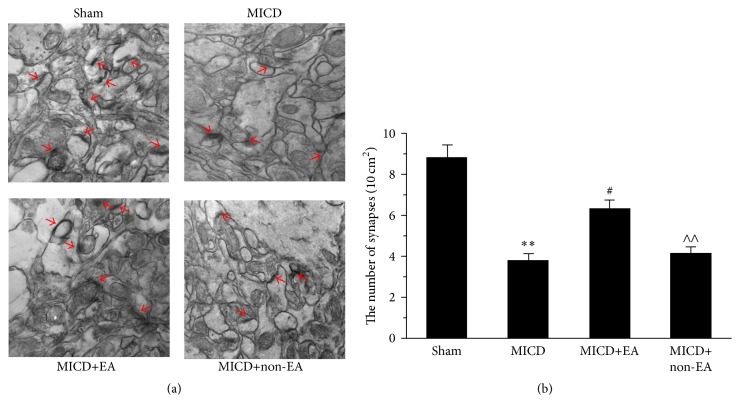
The number of synapses in hippocampal CA1 pyramidal cells. (a) Representative electron micrographic images of synapses (arrows) in the CA1 region of each group (magnification, ×50000). (b) Histogram shows the significant difference in the mean ± SEM of the number of synapses in each group (*n* = 5/group; ^*∗∗*^ *p* < 0.01 versus the Sham group; ^##^ *p* < 0.01, ^#^ *p* < 0.05 versus the MICD group; ^∧∧^ *p* < 0.01, ^∧^ *p* < 0.05 versus the MICD+EA group).

Furthermore, we observed the effect of EA on ultrastructural morphology of hippocampal CA1 pyramidal neurons. Obviously, the density of synapses in the MICD group was decreased compared with the Sham group (*p* < 0.01, Figures 5(a) and 5(b)), whereas there was amplifying in the number of synapses in the MICD+EA group compared with the MICD group and the MICD+non-EA group (*p* < 0.05 or *p* < 0.01, Figures 5(a) and 5(b)).

Taken together, these results suggest that EA could improve synaptic-dendritic plasticity in vivo.

### 3.5. EA Promoted LIMK1 Expression and Phosphorylation of Hippocampus CA1 in MICD Rats

**Figure 6:**
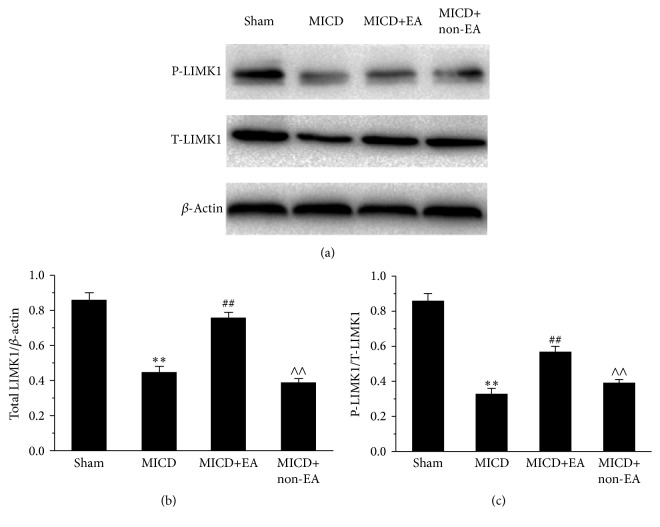
EA amplified the LIMK1 level in the hippocampus CA1 pyramidal cells. (a) The expression of total LIMK1 and phosphorylated LIMK1 was evaluated using western blotting. (b and c) Histogram shows significant difference of the levels of P-LIMK1 and phosphor-LIMLK1 in each group. Data are means ± SEM (*n* = 6/group; ^*∗∗*^ *p* < 0.01 versus the Sham group; ^##^ *p* < 0.01, ^#^ *p* < 0.05 versus the MICD group; ^∧∧^ *p* < 0.01, ^∧^ *p* < 0.05 versus the MICD+EA group).

To explore the underlying molecular mechanism of EA-induced synaptic-dendritic plasticity, the levels of total LIMK1 and phospho-LIMK1 (P-LIMK1, Thr508) in hippocampal CA1 were investigated. As shown in Figures 6(a) and 6(b), the expression of total LIMK1 of hippocampal CA1 was significantly decreased in the MICD group compared with the Sham group (*p* < 0.01). However, the total LIMK1 level in the MICD+EA group was more than that of the MICD group and the MICD+non-EA group (*p* < 0.01, Figures 6(a) and 6(b)). Moreover, the changes of phospho-LIMK1 (Thr508) in groups were similar to the total LIMK1 expression. The level of P-LIMK1 was significantly increased in the MICD+EA group compared with the MICD group and the MICD+non-EA group (*p* < 0.01, Figures 6(a) and 6(c)).

### 3.6. EA Regulated miR-134 Expression in Hippocampal CA1 in MICD Rats

**Figure 7:**
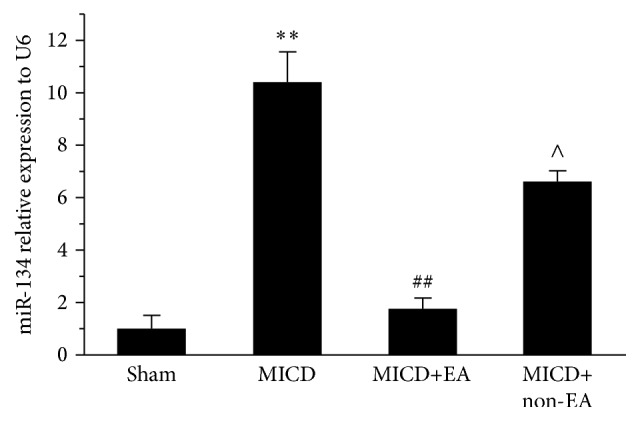
Changes of miR-134 expression in the hippocampus CA1 cells. The changes of miR-134 expression level were measured using quantitative RT-PCR. U6 was used as internal control for quantification of miRNAs. Data are mean ± SEM (*n* = 6/group; ^*∗∗*^ *p* < 0.01 versus the Sham group; ^##^ *p* < 0.01, ^#^ *p* < 0.05 versus the MICD group; ^∧∧^ *p* < 0.01, ^∧^ *p* < 0.05 versus the MICD+EA group).

To identify the role of synaptic-dendrite-related miR-134 in hippocampus CA1, the levels were detected. As shown in Figure 7, the expression of miR-134 was significantly increased in the MICD group compared with the Sham group (*p* < 0.01). However, repeated EA treatment significantly decreased the expression of miR-134 compared with the MICD group and the MICD+non-EA group (*p* < 0.05).

## 4. Discussion

Ischemic stroke leads to a high incidence of long-term cognitive impairment, which is strongly associated with loss of hippocampal neurons and synapses [34]. While reviewing ancient Chinese documents regarding acupuncture and cognitive impairment-related terms, we discovered that the DU20 and DU24 acupoints were the most frequently selected acupoints for cognitive impairment-related rehabilitation in China. A number of studies have shown that EA can improve learning and memory ability and stimulate consciousness [30, 31, 35]. These results indicated that EA could be a complementary therapy for cognitive impairment after stroke. The present study found that EA at the DU20 and DU24 acupoints shortened time to find the platform and increased the times of crossing the position of the platform compared to untreatment or nonacupoint EA (stimulation control) in Morris water maze test, suggesting that EA could improve spatial reference learning and memory ability in MCAO-induced cognitive deficit (MICD) rats. Moreover, it is worth mentioning that the path length in the Morris water maze in the four groups showed no significant difference, indicating that the time spending of MICD rats to find the object was not affected by the motor function. However, the mechanisms of cognitive treatment involved are far from being fully elucidated.

Firstly, the cognitive deficit-related brain regions were determined by a small animal MRI in rats with ischemic stroke. T2-weighted imaging showed that MICD caused the lesions of left cortex, hippocampus, corpus striatum, and thalamus regions before EA. It had been noticed that the brain lesion exhibited a mild spontaneous recovery performance in MICD rats. However, the lesion regions comprising approximately 23% of the whole brain volume were reduced to 15% by EA treatment, indicating that EA could attenuate cortex and hippocampus, corpus striatum, and thalamus region lesions in the MICD rats. Some of these regions, such as the hippocampus and cortex, are essential for regulating learning and memory behaviors including spatial exploration [36]. Studies have confirmed that the hippocampus regions play a very important role in learning and memory through their specific structure and location which connect with other brain regions together [37]. Furthermore, in order to identify the hippocampus function in cognitive deficit, the hippocampal morphology staining was observed.

Using Golgi staining and electron microscopy, we demonstrated that the density of dendritic spine and the number of synapses in hippocampal CA1 pyramidal cells were obviously reduced at Day 14 after MICD. However, EA can rescue the loss of dendritic spine and synapses in hippocampal CA1 region. Moreover, EA promoted synaptic-dendritic spine regeneration. Dendritic spine is the tiny protrusions with multi-dense body and ion channels on the surface of various types of neurons, which serve as cellular substrates of brain connectivity and the major sites of information processing in the brain [38, 39]. Evidence is increasing that synaptic-dendritic plasticity enables the encoding of memory [40]. Indeed, the modification on dendritic spine number, size, and shape is of importance for the plasticity of synapses, accompanied by hippocampus-dependent learning and memory [41, 42]. Studies have well described that dendritic spine remodeling in ischemia surrounding areas may contribute to cognitive functional recovery after stroke [43, 44].

Increasing evidence showed that LIMK1 protein is activated at dendritic spine membranes [45]. LIMK1 positive cells were located in the CA1 region of the hippocampus, which is important in the regulation of the spine morphology and synaptic function in vivo. A prominent form of long lasting synaptic plasticity is thought to be critical to memory formation [26]. The present study found that the expression of total LIMK1 and phospho-LIMK1 in hippocampal CA1 was significantly decreased in the MICD rats; however, EA could enhance the total LIMK1 and phospho-LIMK1 levels to promote synaptic-dendritic plasticity in hippocampal CA1 area.

In addition, we have identified a dendritically localized miRNA that regulated the expression of the synaptic LIMK1 protein, thereby controlling dendritic pine size. miR-134 is the first discovered dendritic microRNA, enriched in the neuronal dendrites of rat hippocampal neurons, and negatively controls the size of dendritic spines [46]. This effect is mediated by miR-134 inhibition of the translation of LIMK1 mRNA [47]. The present study showed that the expression of miR-134 was obviously increased in the MICD rats. However, repeated EA treatment could relieve the upregulation of miR-134 expression in hippocampal CA1 area.

In conclusion, the study indicated that EA at the DU20 and DU24 acupoints could ameliorate cognitive impairment of MICD rats through the regulation of synaptic-dendritic plasticity of the hippocampal CA1 area, and the expression and phosphorylation of synaptic LIMK1 protein were activated to control dendritic spine number, size, and shape. Furthermore, the mechanism of LIMK1 increase induced by EA treatment may be associated with miR-134, which is localized in hippocampal CA1, by negatively regulating LIMK1 to enhance synaptic-dendritic plasticity in the recovery stage of ischemic stroke.
